# Genome-Wide Analysis of the Universal Stress Protein Gene Family in Blueberry and Their Transcriptional Responses to UV-B Irradiation and Abscisic Acid

**DOI:** 10.3390/ijms242316819

**Published:** 2023-11-27

**Authors:** Yan Song, Bin Ma, Xinghua Feng, Qingxun Guo, Lianxia Zhou, Xinsheng Zhang, Chunyu Zhang

**Affiliations:** College of Plant Science, Jilin University, Changchun 130062, China

**Keywords:** *VcUSPs*, blueberry, UV-B radiation, ABA, RNA-seq, WGCNA

## Abstract

Universal stress proteins (USPs) play essential roles in plant development, hormonal regulation, and abiotic stress responses. However, the characteristics and functional divergence of USP family members have not been studied in blueberry (*Vaccinium corymbosum*). In this study, we identified 72 *VcUSP* genes from the Genome Database for *Vaccinium*. These *VcUSPs* could be divided into five groups based on their phylogenetic relationships. VcUSPs from groups Ⅰ, Ⅳ, and Ⅴ each possess one UspA domain; group Ⅰ proteins also contain an ATP-binding site that is not present in group Ⅳ and Ⅴ proteins. Groups Ⅱ and Ⅲ include more complex proteins possessing one to three UspA domains and UspE or UspF domains. Prediction of cis-regulatory elements in the upstream sequences of *VcUSP* genes indicated that their protein products are likely involved in phytohormone signaling pathways and abiotic stress responses. Analysis of RNA deep sequencing data showed that 21 and 7 *VcUSP* genes were differentially expressed in response to UV-B radiation and exogenous abscisic acid (ABA) treatments, respectively. *VcUSP41* and *VcUSP68* expressions responded to both treatments, and their encoded proteins may integrate the UV-B and ABA signaling pathways. Weighted gene co-expression network analysis revealed that *VcUSP22*, *VcUSP26*, *VcUSP67*, *VcUSP68*, and *VcUSP41* were co-expressed with many transcription factor genes, most of which encode members of the MYB, WRKY, zinc finger, bHLH, and AP2 families, and may be involved in plant hormone signal transduction, circadian rhythms, the MAPK signaling pathway, and UV-B-induced flavonoid biosynthesis under UV-B and exogenous ABA treatments. Our study provides a useful reference for the further functional analysis of *VcUSP* genes and blueberry molecular breeding.

## 1. Introduction

Universal stress proteins (USPs) are members of the adenine nucleotide alpha hydrolase (AANH) superfamily (PF00582 protein family in the Pfam classification) that are widely conserved in bacteria, archaea, plants, and metazoans [1]. There are six USPs (UspA, UspC, UspD, UspE, UspF, and UspG) in *Escherichia coli*; *UspA* was the first of these genes to be cloned and sequenced [2,3,4,5]. The USP domain has the capacity to form homodimers and heterodimers. USPs contained a single USP domain or two tandem repeats of USP domains or a USP domain alongside other functional domains [6,7]. The USP domain contains 130–160 highly conserved amino acid residues and can be classified into two categories based on the presence or absence of the ATP-binding site G-2X-G-9X-G-(S/T) [8,9,10]. USPs participate in responses to a multitude of starvation and stress stimuli [5,11].

The first plant USP was identified in rice (*Oryza sativa*). Many USPs have been characterized in a variety of plant species, including *Arabidopsis thaliana*, wild tomato (*Solanum pennellii*), wild apple (*Malus sieversii*), and grapevine (*Vitis vinifera*) [12,13,14,15,16]. USPs respond to abiotic stress (salt, drought, cold, heat, UV-B, wounding, and osmotic stress) in plants and are also involved in plant hormone signaling pathways, including abscisic acid (ABA), gibberellin (GA), and ethylene signaling [12,15,17,18,19]. Most *AtUSPs* in *Arabidopsis* are induced by UV-B treatment, while most *VvUSPA* genes in grapevine show transcriptional responses to ABA [19,20]. In Arabidopsis, USPs regulate the circadian rhythm of the central oscillator genes *CIRCADIAN CLOCK ASSOCIATED 1 (CCA1) and TIMING OF CAB EXPRESSION 1* (*TOC1*) [21]. Various USPs interact with other proteins to mediate stress tolerance [17]; for example, SpUSPs in wild tomato may interact with annexin to increase drought tolerance in the seedling and adult stages by influencing ABA-induced stomatal movement, increasing photosynthesis, and alleviating oxidative stress [22]. VyUSPA3 enhances drought tolerance in Chinese wild grape (*Vitis yeshanensis*), possibly by interacting with phytohormone signaling pathways, the ubiquitination system, ethylene-responsive element binding factors, or nuclear factors [16].

Plants are subjected to a variety of abiotic stresses. UV-B radiation is an environmental signal that affects plant growth and development and controls many processes involved in physiological and biochemical acclimation. The phytohormone ABA modulates physiological processes by controlling plant responses to biotic and abiotic stress. Several studies have explored the interaction between UV-B and ABA signaling [23,24,25]. ABA treatment increased the tolerance of grapevine leaves to UV-B, while phenol levels in berry skins additively increased, including changes in anthocyanin and non-anthocyanin profiles, under both UV-B and ABA treatments [23,26]. However, it is not clear which genes integrate the UV-B and ABA signaling pathways.

Blueberry (*Vaccinium corymbosum*) is an economically important small fruit crop worldwide due to the high phenolic acid and flavonoid contents of its fruits and leaves [27,28]. Both UV-B and ABA promote flavonoid accumulation in blueberry fruit by regulating the expression of genes encoding MYB transcription factors and proteins involved in flavonoid biosynthesis [29,30,31,32]. Although *USP* genes have been identified in Arabidopsis, barley (*Hordeum vulgare*), grapevine, and rice, a comprehensive study of *USP* genes in blueberry has not yet been reported [19,33,34,35,36]. The sequencing and assembly of the *V. corymbosum* cv. Draper genome was completed, and its function was annotated in March 2019. This Genome Database offers the possibility to systematically identify and investigate the putative functions of USP family members in blueberry because of its high-quality data [37]. A recent study showed that VcUSP1 responds to UV-B radiation [38]; however, the roles of all VcUSP family members in UV-B and ABA responses remain unknown.

In this study, we identified 72 *VcUSP* genes from the Genome Database for *Vaccinium* and predicted their gene structures, evolutionary relationships, and conserved motifs and domains of both the encoded proteins and the *cis*-regulatory elements in the promoters. We analyzed the responses of *VcUSP* genes to UV-B radiation and ABA treatments based on RNA deep sequencing (RNA-seq) data and examined the relationships between *VcUSPs* and transcription factors under UV-B and ABA treatments via weighted gene co-expression network analysis (WGCNA). Our results provide valuable information for the further functional characterization of blueberry *VcUSP* genes and guidance for blueberry breeding under UV-B radiation and ABA treatments.

## 2. Results

### 2.1. Identification of VcUSP Gene Family Members in the Blueberry Genome

**Figure 1:**
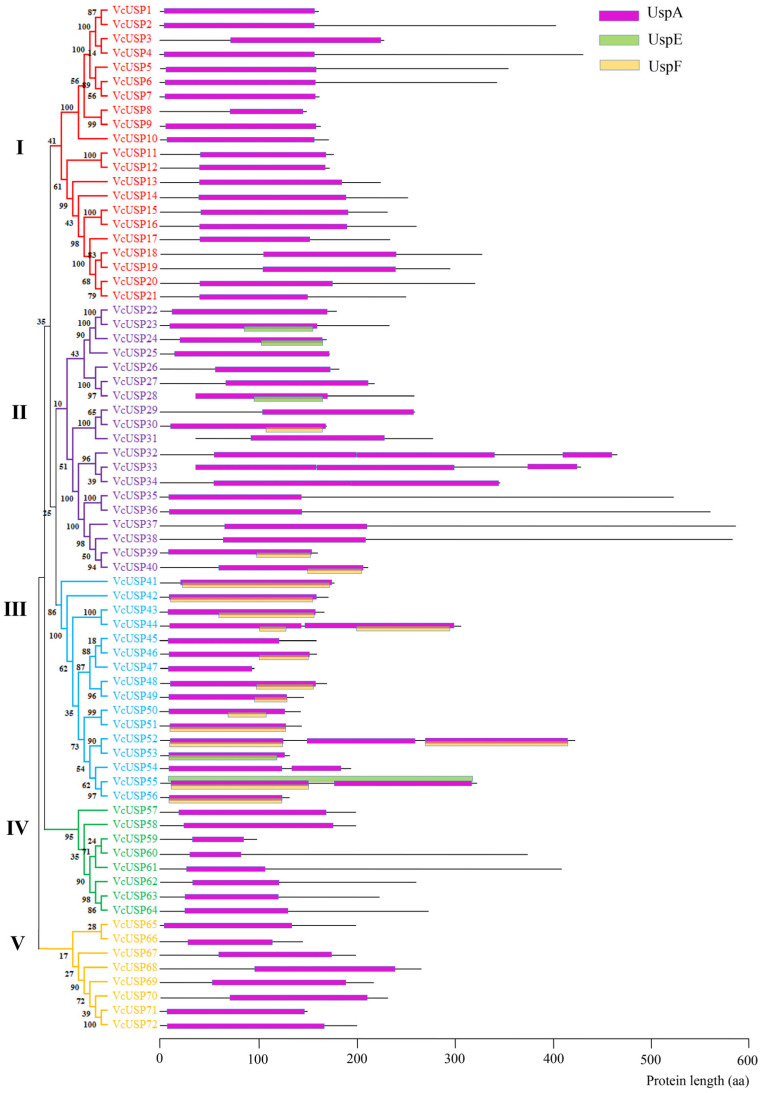
Phylogenetic relationships and USP domains of blueberry USPs. Different font colors represent the different USP groups. Red, pruple, blue, green and yellow VcUSPs were clusted in groups I, II, III, IV and V, respecteively.

To identify putative *VcUSPs*, we conducted a Hidden Markov Model (HMM) search using the USP domain (PF00582) as a query against the Genome Database for *V. corymbosum* cv. Draper V1.0. After removing sequences without USP or AANH domains and short and redundant sequences, 72 putative *USP* family genes were identified in blueberry (Supplementary Table S1). We named these *USP* genes *VcUSP1* to *VcUSP72* based on evolutionary analysis (Figure 1). The VcUSP proteins ranged from 150 to 587 amino acids in length and possessed a UspA domain; some also contained UspE or UspF domains (Table 1). The molecular weights of the VcUSPs ranged from 16.22 to 63.69 kDa, and the theoretical isoelectric point (pI) ranged from 4.62 to 10.51.

### 2.2. Phylogenetic Analysis of the VcUSP Family 

To investigate the evolutionary relationships of the 72 VcUSP family members, we constructed a phylogenetic tree based on their deduced amino acid sequences (Figure 1). The VcUSPs were categorized into five groups. VcUSPs in group Ⅰ (VcUSP1–21), group Ⅳ (VcUSP57–65), and group Ⅴ (VcUSP65–72) only contained one UspA domain, while those in group Ⅱ (VcUSP22–40) and group Ⅲ (VcUSP41–56) possessed one to three UspA domains, with some also possessing UspE or UspF domains. VcUSP23, VcUSP24, VcUSP28, and VcUSP53 contained a UspE domain, while 14 VcUSPs (VcUSP30, VcUSP39–44, VcUSP46, VcUSP48–52, and VcUSP56) contained a UspF domain. VcUSP55 contained both a UspE domain and a UspF domain. The 53 other VcUSPs contained only a UspA domain.

**Figure 2:**
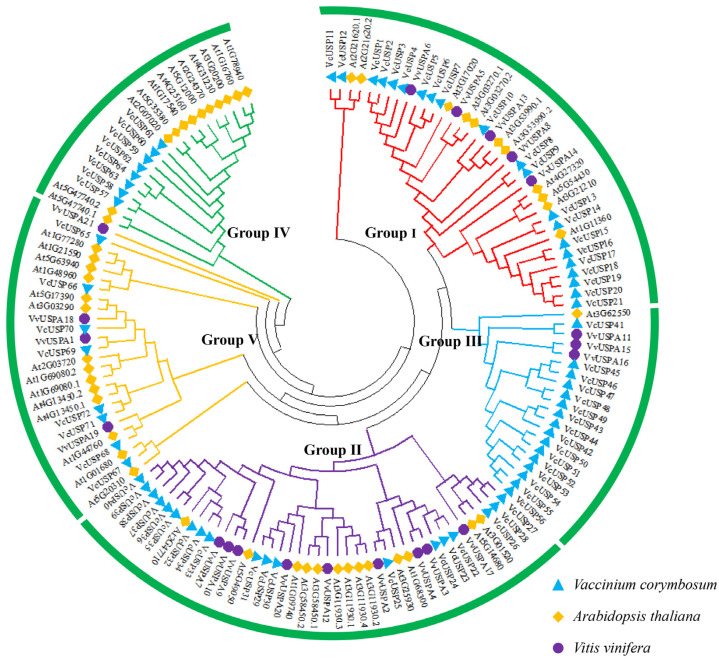
Phylogenetic analysis of USPs in blueberry (*Vaccinium corymbosum*), Arabidopsis (*Arabidopsis thaliana*), and grapevine (*Vitis vinifera*). Different colors represent the different USP groups.

We constructed another phylogenetic tree of USPs from blueberry (72 members), Arabidopsis (51 members), and grapevine (21 members). These USPs were categorized into the five blueberry USP groups (Figure 2). Most VcUSPs were distributed in group Ⅰ (21), with 19 VcUSPs in group Ⅱ, 16 in group Ⅲ, 8 in group Ⅳ, and 8 in group Ⅴ. Among the 21 VvUSPs, 9 belong to group Ⅱ, 5 to group Ⅰ, 4 to group Ⅴ, and 3 to group Ⅲ. Most of the Arabidopsis USPs were distributed in group Ⅳ (14), group Ⅱ (13), group Ⅴ (12), and group Ⅰ (11), and only one AtUSP belonged to group Ⅲ. Many USP family members from blueberry, Arabidopsis, and grapevine were clustered in the same groups, suggesting that VcUSPs share similar functions with USPs from other plant species.

### 2.3. Multiple Sequence Alignment of VcUSPs

**Figure 3:**
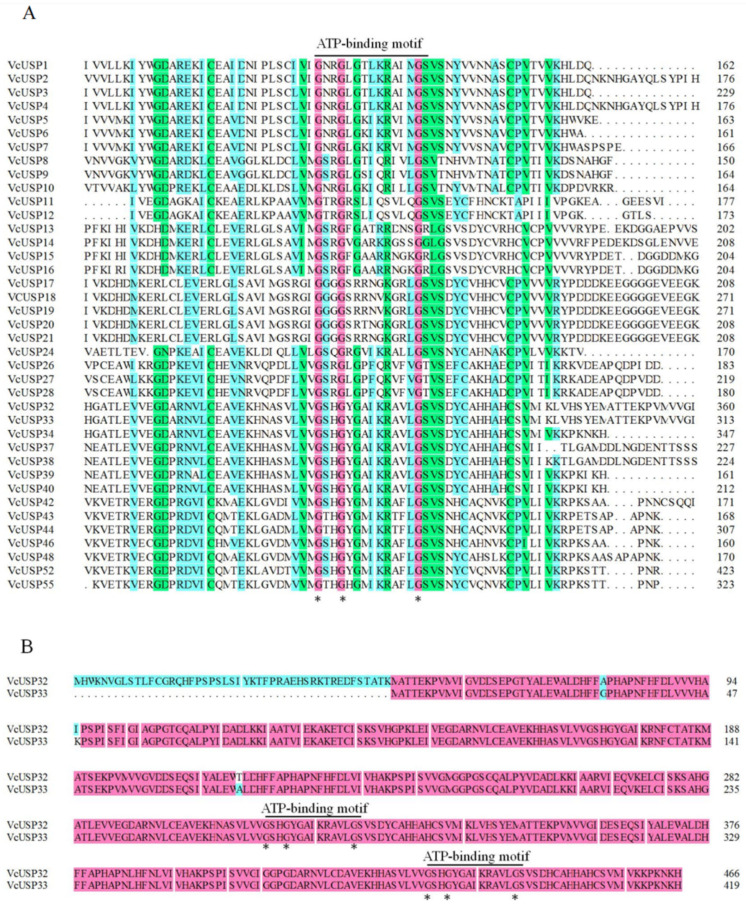
Amino acid sequence alignment of VcUSP proteins. (**A**) Amino acid sequence alignment of proteins with one ATP-binding motif. (**B**) Amino acid sequence alignment of proteins with two ATP-binding motifs. The asterisks indicate the predicted ATP-binding site G-2X-G-9X-G-(S/T). The pink background indicates 100% conservation; green indicates ≥ 75% conservation; blue indicates ≥ 50% conservation.

USPs can be classified as ATP binding or non-ATP binding based on the presence or absence of ATP-binding sites [8,9,10]. Sequence alignment showed that 39 VcUSPs contained an ATP-binding site, with VcUSP32 and VcUSP33 possessing two ATP-binding sites (Figure 3A,B). All group Ⅰ VcUSPs contained a single ATP-binding site, while all group Ⅳ and Ⅴ VcUSPs lacked an ATP-binding site. Of the 19 VcUSPs in group Ⅱ, 11 contained an ATP-binding site, while 7 of the 15 in group III VcUSPs contained an ATP-binding site (Table 1).

We also analyzed the UspA domains of the VcUSPs via sequence alignment (Supplementary Figure S1). Of the 72 VcUSPs, 65 contained one UspA domain (Supplementary Figure S1A); VcUSP34, VcUSP44, VcUSP54, and VcUSP55 contained two UspA domains (Supplementary Figure S1B); and VcUSP32, VcUSP33, and VcUSP52 contained three UspA domains (Supplementary Figure S1C). Group Ⅰ, Ⅳ, and Ⅴ VcUSPs all contained only one UspA domain, while those in groups Ⅱ and Ⅲ contained one, two, or three UspA domains. These results indicate that VcUSP from groups Ⅱ and Ⅲ have more complex structures than those from groups Ⅰ, Ⅳ, and Ⅴ.

### 2.4. Gene Structures of VcUSPs

**Figure 4:**
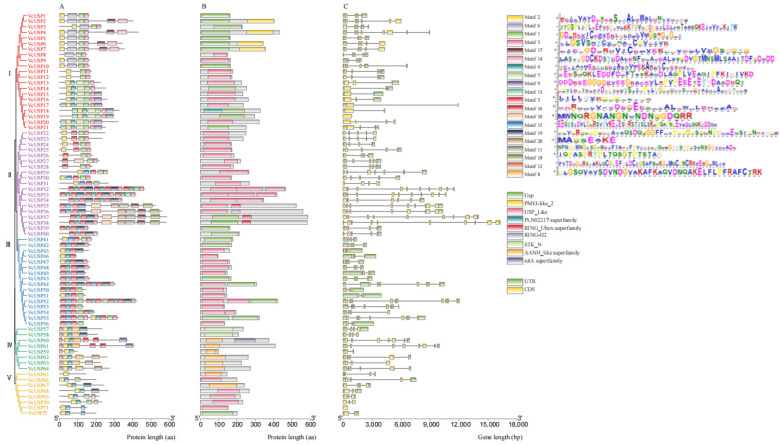
Diagrams of the motif compositions, conserved domains, and gene structures of VcUSPs. (**A**) Motif compositions of VcUSPs. The maximum number of motifs was set to 20. Different colored boxes represent the corresponding conserved motifs on the upper right. (**B**) The conserved domains of VcUSPs. Different colored boxes represent the corresponding conserved domains on right center. (**C**) Structures of the *VcUSP*s. Dark green boxes, orange boxes, and black lines represent UTRs, exons, and introns, respectively. Red, pruple, blue, green and yellow VcUSPs were clusted in groups I, II, III, IV and V, respecteively. The sequence lengths of each protein and gene are represented by gray bars at the bottom.

To gain further insight into the structural characteristics of *VcUSP* genes, we predicted the presence of 20 conserved motifs in these genes using the MEME website (Figure 4A). These motifs were distributed across different *VcUSP*s, with the highest number (13) found in *VcUSP32*, *VcUSP33*, and *VcUSP52*; *VcUSP65* contained only one motif. All *VcUSPs* from group Ⅰ contained conserved motifs 1, 2, and 3; most *VcUSP*s from groups Ⅱ and Ⅲ contained conserved motifs 3, 5, 6, and 16; and most *VcUSP*s from groups Ⅳ and Ⅴ contained conserved motifs 2 and 4. Conserved motifs 6, 7, 13, and 14 were only observed in group Ⅰ, while motifs 10, 11, and 18 were found only in group Ⅱ. Conserved motifs 8 and 12 were detected only in group Ⅳ, while motif 15 was found in groups Ⅱ and Ⅲ. *VcUSP*s that clustered in the same groups shared a similar motif pattern. Conserved motifs 1, 2, 3, and 4 were present in all groups; therefore, we considered these to be the main motifs of the *VcUSP* family.

In addition to the USP domain, we also identified six other domains in VcUSPs (Figure 4B). In group I, the plant invertase/pectin methylesterase inhibitor (PMEI-like_2, cd15800) domain was observed near the C-termini of VcUSP2, VcUSP4, VcUSP6, and VcUSP7, while the pectinesterase/pectinesterase inhibitor (PME, PLN02217) domain was present near the N-terminus of VcUSP18. The conserved motifs 7 and 14 are related to PMEI-like_2. In group II, the RING finger domain and U-box domain superfamily (RING_Ubox, cl17238) was found near the C-termini of VcUSP35, VcUSP37, and VcUSP38, while the H2 subclass of RING finger (RING-H2, cd16448) domain was found near the C-terminus of VcUSP36; conserved motif 10 is related to both RING_Ubox and RING-H2. In group Ⅳ, VcUSP57 and VcUSP58 contained the N-terminal domain of eukaryotic serine threonine kinases (STK_N, cd01989) in the same position as the USP domain (in the middle of the coding sequence), while the C-terminus of VcUSP60 contained the cell envelope integrity inner membrane protein (tolA, PRK09510) domain. These results highlight the complexity and diversity of the VcUSP protein structures.

We also characterized the exon/intron structures within the *VcUSP* genes (Figure 4C). *VcUSP* genes all possessed between 1 and 11 exons; group Ⅰ *VcUSP* genes contained 1–8 exons, group Ⅱ genes contained 4–11 exons, group Ⅲ genes contained 3–8 exons, group Ⅳ genes contained 2–8 exons, and group Ⅴ genes contained 1–4 exons. Among the 72 *VcUSP* genes, 26 contained four exons, 15 contained three exons, and *VcUSP32*–*38* in group Ⅱ contained 7–11 exons. Overall, most VcUSPs harbored three or four exons, and the gene structures of group Ⅱ VcUSPs were the most complex.

### 2.5. cis-Regulatory Elements of VcUSPs 

**Figure 5:**
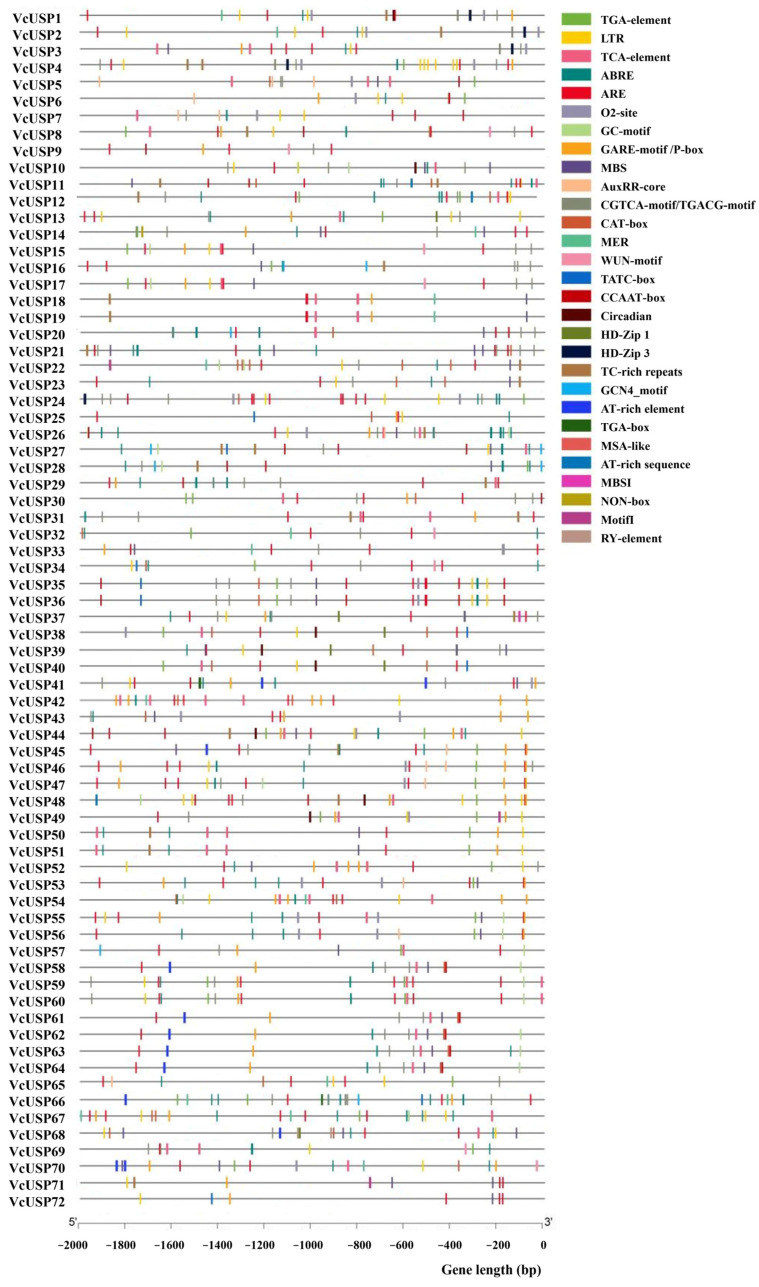
Diagram of the predicted *cis*-regulatory elements in the *VcUSP* promoters. Different colored symbols represent *cis*−regulatory elements, as shown to the right of the diagram.

To predict the potential functions of *VcUSP*s, we analyzed their promoter sequences (2000 bp upstream from the ATG start codon) by predicting and visualizing the *cis*-regulatory elements in these regions (Supplementary Table S2; Figure 5). In addition to *cis*-acting elements related to plant growth and development, we also found environmental stress-responsive elements, including those responsive to light (G box, GTGGC motif, MRE, Sp1, TCCC motif, and TCT motif), low temperature (LTR), drought (MBS), wounding (WUF motif), and biotic defense (TC-rich repeats). We also identified phytohormone-responsive elements, such as those responsive to auxin (TGA element, AuxRR core, and TGA box), GA (GARE motif, P box, and TATC box), salicylic acid (TCA element), jasmonate (CGTCA motif and TGACG motif), and ABA (TCA element). Light-responsive elements were present in the promoters of all VcUSPs, while the ABA-responsive element was identified in the promoters of 56 *VcUSP*s, suggesting that most *VcUSP*s are involved in plant responses to light and ABA.

### 2.6. VcUSP Expression Patterns in Response to UV-B Radiation

**Figure 6:**
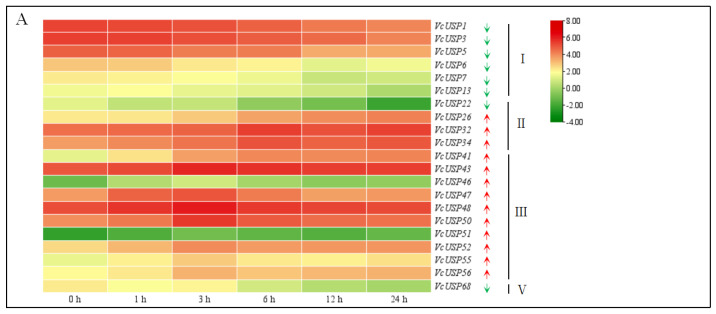

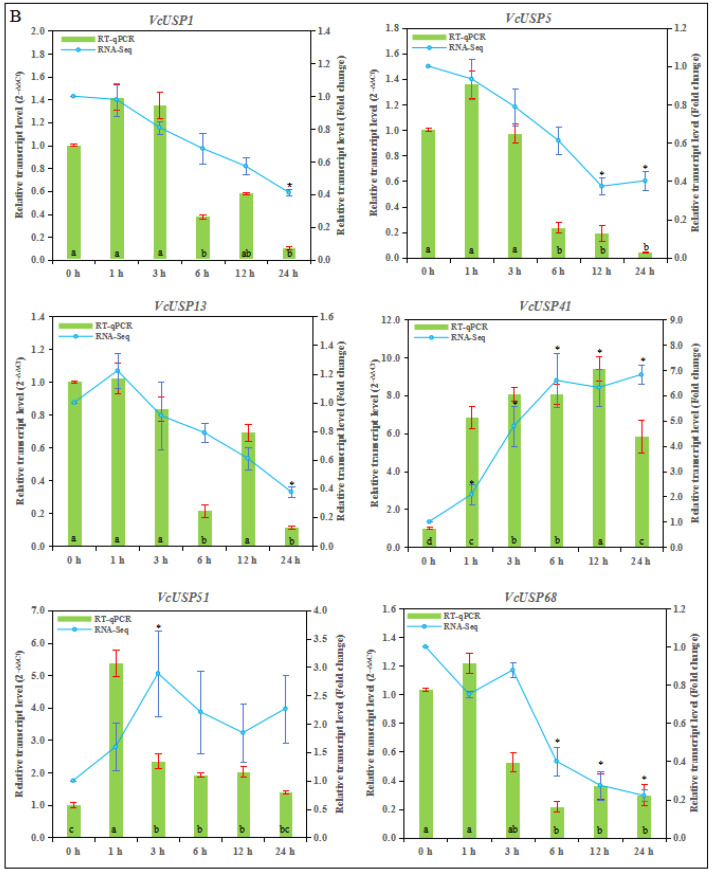
Expression analysis of VcUSPs under UV-B radiation. (**A**) Transcript profiling of *VcUSP*s under UV−B radiation based on log10 (FPKM) values from RNA−seq data. In the color scale, green indicates a low expression level while red indicates a high expression level. Upward and downward arrows represent *VcUSP*s that are upregulated and downregulated by UV−B radiation, respectively. (**B**) Expression patterns of *VcUSP*s under UV-B radiation determined using RT-qPCR and RNA−seq data. Values are means ± SD from three independent biological replicates. Statistically significant differences were determined using Tukey’s test at *p* value ≤ 0.05. The red error bars and blue error bars represent the SD of the samples for RT−qPCR and RNA−seq analysise, respectively. The asterisks (RNA−seq) and different letters (RT−qPCR) indicate significant differences compared with the 0 h control.

To explore the expression patterns of *VcUSP*s in response to light stress, we downloaded RNA-seq data from blueberry calli after 0, 1, 3, 6, 12, and 24 h of UV-B treatment from the BioProject database (Supplementary Table S3). Twenty-one *VcUSP*s were differentially expressed in response to UV-B radiation, including fourteen that were upregulated and seven that were downregulated. Of these differentially expressed genes (DEGs), all six *VcUSP*s from group I (*VcUSP1*, *VcUSP3*, *VcUSP5–7*, and *VcUSP13*), *VcUSP22* from group Ⅱ, and *VcUSP68* from group Ⅴ were downregulated, while 10 *VcUSP* genes from group III (*VcUSP41*, *VcUSP43*, *VcUSP46–48*, *VcUSP50–52*, and *VcUSP55–56*) and three VcUSP genes from group II (*VcUSP26*, *VcUSP32*, and *VcUSP34*) were upregulated by UV-B treatment (Figure 6A; Supplementary Table S3). Overall, UV-B treatment mainly repressed the expression of group Ⅰ genes and promoted the expression of group Ⅲ genes.

To validate the accuracy and reliability of the *VcUSP* gene expression patterns determined based on RNA-seq data, we subjected six differentially expressed *VcUSP*s to RT-qPCR analysis (Figure 6B). The expression levels of *VcUSP1* decreased after 6 h and 24 h of UV-B treatment compared to the control (0 h of UV-B). *VcUSP5*, *VcUSP13*, and *VcUSP68* were also downregulated by UV-B treatment. In contrast, UV-B radiation promoted the expressions of *VcUSP41* and *VcUSP51*. These results verified the accuracy and reliability of the *VcUSP* gene expression patterns determined based on RNA-seq data.

### 2.7. VcUSP Expression Patterns in Response to ABA 

**Figure 7:**
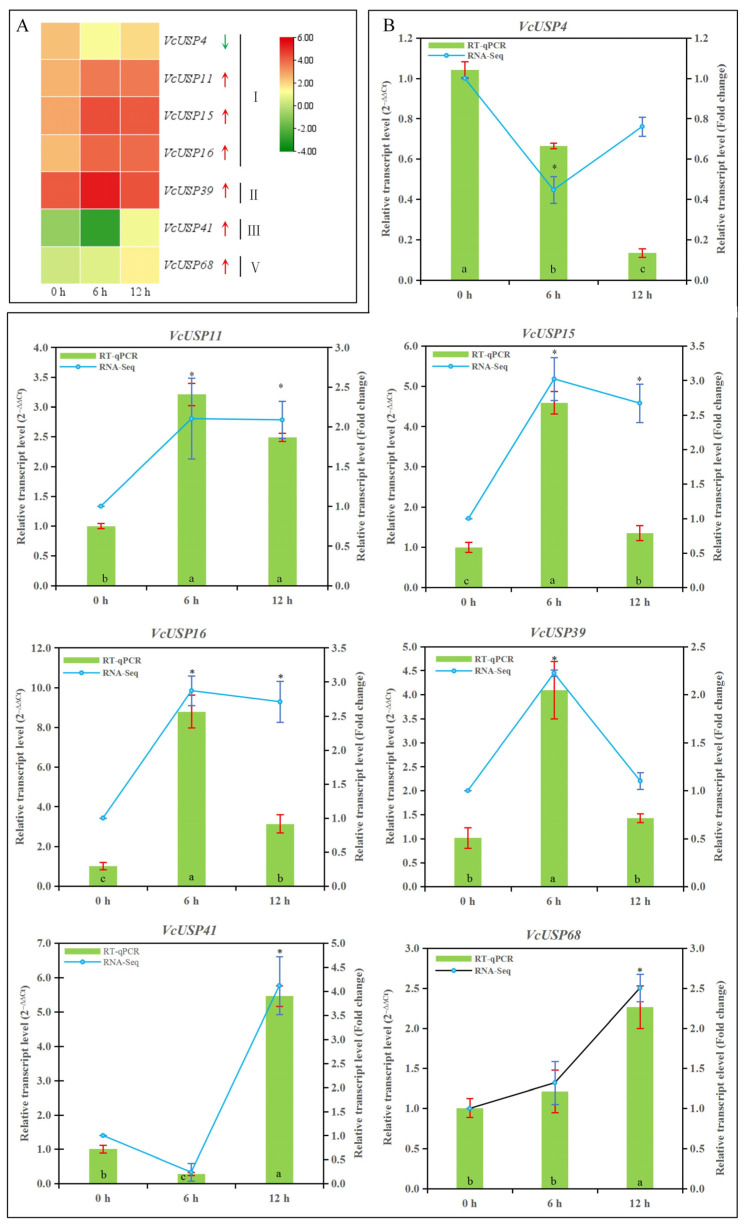
Expression analysis of *VcUSP*s under exogenous ABA treatment. (**A**) Transcript profiling of *VcUSP*s under exogenous ABA treatment based on log10 (FPKM) values from RNA-seq data. In the color scale, green indicates a low expression level while red indicates a high expression level. Upward and downward arrows represent *VcUSP*s that are upregulated and downregulated by exogenous ABA treatment, respectively. (**B**) Expression patterns of *VcUSP*s under exogenous ABA treatment determined using RT-qPCR and RNA-seq data. Values are means ± SD from three independent biological replicates. Statistically significant differences were determined using Tukey’s test at *p* value ≤ 0.05. The red error bars and blue error bars represent the SD of the samples for RT−qPCR and RNA−seq analysise, respectively. The asterisks (RNA-seq data) and different letters (RT-qPCR) indicate significant differences compared with the 0 h control.

We demonstrated that the promoters of *VcUSP* genes contain *cis*-regulatory elements related to phytohormones, especially ABA. Therefore, we downloaded RNA-seq data of in vitro-grown blueberry seedlings subjected to ABA treatment from the BioProject database (Supplementary Table S4). Only seven *VcUSP* DEGs were responsive to 6 h and 12 h of ABA treatment. In group Ⅰ, *VcUSP4* was downregulated by ABA. *VcUSP11*, *VcUSP15*, and *VcUSP16* of group Ⅰ, *VcUSP39* of group Ⅱ, *VcUSP41* of group Ⅲ, and *VcUSP68* of group Ⅴ were upregulated by ABA (Figure 7A). Most of these differentially expressed *VcUSP* genes were from group Ⅰ and were upregulated under ABA treatment.

To confirm the accuracy and reliability of the expression patterns of these seven differentially expressed *VcUSP* genes based on RNA-seq data, we examined their expression using RT-qPCR (Figure 7B). The expression levels of *VcUSP1*1, *VcUSP15*, *VcUSP16*, and *VcUSP39* significantly increased in response to ABA treatment, reaching levels that were 3.2-, 4.6-, 8.8-, and 4.1-fold higher than the control after 6 h of ABA treatment, respectively, while *VcUSP41* showed a significant (5.5-fold) increase in expression after 12 h of ABA treatment. These expression patterns determined using RT-qPCR were similar to those observed in the RNA-seq data, verifying the reliability of the RNA-seq data.

### 2.8. Identification of VcUSPs Co-Expressed with Transcription Factor Genes under UV-B and ABA Treatments Using WGCNA

**Figure 8:**
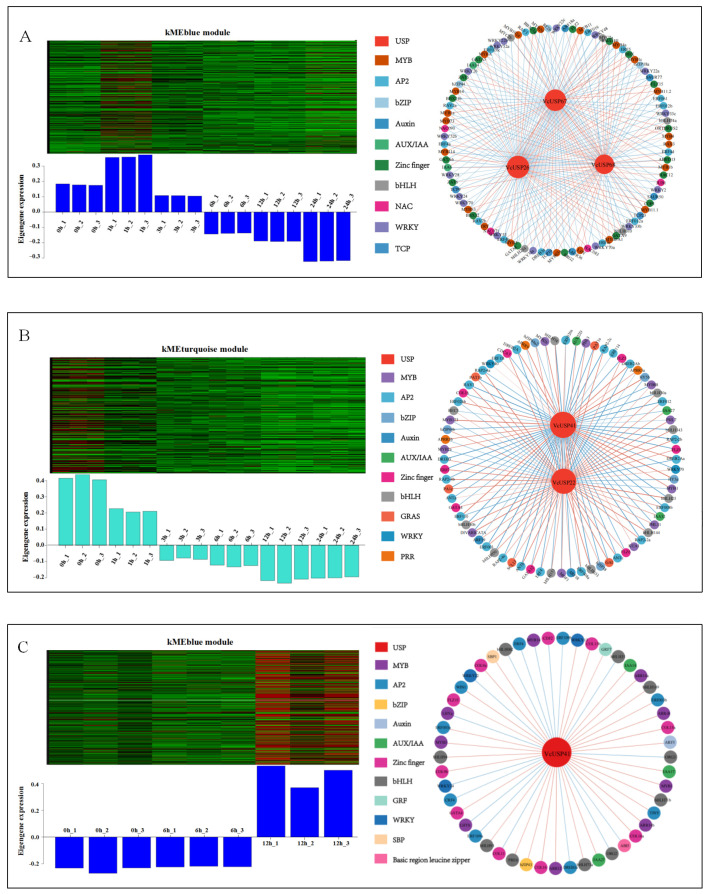
WGCNA module and co-expression network of differentially expressed *VcUSP*s and transcription factor genes under UV-B radiation or exogenous ABA treatment. (**A**) Blue module and the corresponding co−expression network from WGCNA data under UV−B radiation. (**B**) Turquoise module and the corresponding co−expression network from WGCNA data under UV-B radiation. (**C**) Blue module and the corresponding co−expression network from WGCNA data under exogenous ABA treatment. Red represents a high expression level and green represents a low expression level in the heat maps. Each blueberry USP protein and transcription factor family are represented by a box or circle of a different color. Red lines indicate positive correlations; blue lines indicate negative correlations. The thickness of the line represents the degree of correlation. The size of the circle represents the number of related genes.

USPs regulate plant responses to abiotic stress, likely via interactions with transcription factors [16]. To elucidate the interactions of VcUSPs with transcription factors that function in plant responses to UV-B radiation and ABA, we searched for transcription factor genes that were co-expressed with *VcUSPs* using the WGCNA package in R and calculated their Pearson’s correlation coefficients (*r* values). The WGCNA clustered all the DEGs into three modules per treatment: kMEblue, kMEbrown, and kMEturquoise for UV-B radiation and kMEblack, kMEblue, and kMEturquoise for ABA (Supplementary Figure S2). *VcUSPs* were present in the kMEblue and kMEturquoise modules under UV-B treatment and the kMEblue module under ABA treatment (Figure 8A–C; Supplementary Table S5). Thus, we used the kMEblue and kMEturquoise modules (UV-B radiation) and the kMEblue module (ABA treatment) for protein interaction analysis.

We identified transcription factor genes and VcUSPs from the above modules. For the kMEblue module (UV-B treatment), *VcUSP26*, *VcUSP67*, and *VcUSP68* were co-expressed with 89 genes encoding transcription factors from the MYB (26.6% of these genes), WRKY (16.85%), bHLH (5.62%), zinc finger (21.35%), AP2 (15.73%), auxin, AUX/IAA, bZIP, TCP, and NAC families (Supplementary Table S6; Supplementary Figure S3A). For the kMEturquoise module under UV-B treatment, *VcUSP22* and *VcUSP41* were co-expressed with 72 transcription factor genes (Supplementary Table S7), including 33.33% from the AP2 family, 15.28% from the MYB family, 13.89% from the bHLH family, 11.11% from the zinc finger family, and 26.39% from other families (including WRKY, auxin, AUX/IAA, bZIP, GRAS, and PRR; Supplementary Figure S3B). The kMEblue module under ABA treatment contains only *VcUSP41* and *VcUSP49* transcription factor genes, including members of the zinc finger (20.41%), bHLH (20.41%), MYB (18.37%), AP2 (18.37%), and other transcription factor families (auxin, AUX/IAA, bZIP, GRF, and SBP; Supplementary Table S8; Supplementary Figure S3C).

To screen for transcription factors whose expressions are significantly correlated with *VcUSP*s, we calculated Pearson’s correlation coefficients and reconstructed the co-expression networks according to the *r* values (Supplementary Table S9; Figure 8A–C). VcUSPs were positively or negatively correlated with the corresponding transcription factors; for example, *VcUSP67* was positively correlated with most MYB family members and negatively correlated with AP2 family members, while *VcUSP26* was positively correlated with 13 MYB transcription factors and negatively correlated with 8 MYB transcription factors in the kMEblue module under UV-B treatment. Kyoto Encyclopedia of Genes and Genomes (KEGG) pathway analysis showed that most of these transcription factors were involved in hormone signal transduction (ko04075), the circadian rhythm (ko04712), and the MAPK signaling pathway (ko04016). These results indicate that VcUSPs are mainly co-expressed with transcription factors from the MYB, AP2, zinc finger, and bHLH families and that VcUSPs may regulate the expression of downstream genes through synergistic or antagonistic effects with certain transcription factors under UV-B or ABA treatment.

## 3. Discussion

### 3.1. Structural Diversity of the VcUSP Family

USPs are present in a wide variety of organisms and participate in a range of cellular responses to biotic and abiotic stress. To date, 44 *USP* genes have been identified in Arabidopsis, 21 in barley, 21 in grapevine, and 44 in rice [19,34,35,36]. In the present study, we identified 72 *VcUSP* genes in blueberry. This high number relative to Arabidopsis and other previously characterized plant species may be related to genome duplication in blueberry or to differences in genome size or evolutionary histories between the species [37,39].

We determined that VcUSPs can be structurally diverse. USPs in other species generally contain a single UspA domain or two tandem repeats of UspA domains [3]. Most VcUSPs contained one or two UspA domains; however, several VcUSPs contained three tandem repeats of UspA domains. Some VcUSPs contained not only UspA domains but also UspE and/or UspF domains; VcUSP55 contained two UspA domains, one UspE domain, and one UspF domain. In bacteria and plants, USPs contain other domains in addition to USP domains; for example, protein kinase-like, TPR-like, ApoLplll-like, U-box, and CDC37_N_like domains are present in UspA proteins in Madagascar periwinkle (*Catharanthus roseus*), while pkinase, pkinanse_Try, and U-box domains are found in Arabidopsis and rice USPs [36,40]. In this study, we determined that six domains (PMEI-like_2, PME, RING_Ubox, RING-H2, STK_N, and tolA) coexisted with the USP domains in VcUSPs. PMEI and PME are the main enzymes acting on pectin, a major component of the plant cell wall [41]. Most proteins containing RING_Ubox and RING-H2 domains are E3 ubiquitin ligases with a variety of cellular functions, including development, signal transduction, and stress responses [42,43,44]. The N-terminal domain STK_N is homologous to the ATP-binding fold in the USP family [45]. The TolA protein is involved in maintaining the integrity of the outer membrane [46]. These domains endow the VcUSP proteins a variety of functions.

To further elucidate the structural characteristics of the blueberry *VcUSP*s, we analyzed their conserved motifs and exon–intron structures. We identified 20 motifs, with motifs 1–11 found in each of the *VcUSP*s. Some motifs (such as motifs 6–8, 10–14, and 18) were only present in one VcUSP group. The number of exons in *VcUSP*s ranged from 1 to 11 per gene (Figure 4). Only four motifs are present in *VvUSP*s from grapevine, and all *VvUSP*s contain two to four introns [19]. The 44 rice *OsUSP* genes each contain 3–10 motifs and show moderate variation in terms of the number of exons, ranging from 1 to 11 [36]. Most *USP*s in Arabidopsis and barley contain two to four exons [35]. These results indicate that VcUSPs are more variable than USPs of other species in terms of both their conserved motifs and exon–intron structures.

### 3.2. The Evolutionary Relationships of VcUSPs

Our phylogenetic analyses classified all VcUSPs into five subgroups, which is consistent with previous findings in grapevine, Arabidopsis, and barley [19]. All VcUSPs from groups Ⅰ, Ⅳ, and Ⅴ contained only one UspA domain but no UspE or UspF domains. The UspA domains were further subdivided based on whether they contained an ATP-binding site: VcUSPs from group Ⅰ contained a UspA ATP-binding site, but all VcUSPs from groups Ⅳ and Ⅴ did not (Table 1; Figure 1). Therefore, it appears that group Ⅰ *VcUSP*s originated in different branches of the evolutionary tree compared to groups Ⅳ and Ⅴ VcUSPs. On the contrary, the components of group Ⅱ and Ⅲ were complex, with some VcUSPs containing one, two, or three UspA domains and UspE or UspF domains; VcUSPs with or without ATP-binding sites were also clustered into two groups (Table 1; Figure 1). Our findings suggest that groups Ⅱ and Ⅲ underwent rapid expansion, while groups Ⅰ, Ⅳ, and Ⅴ underwent a rapid loss of *VcUSP*s. These conclusions are supported by the conserved motifs and exon–intron structures of these genes, in that the genes of groups Ⅳ and Ⅴ had more complex and diverse structures (Table 1; Figure 1). Figure 4 shows that USPs from blueberry and Arabidopsis were distributed in each phylogenetic group, pointing to similar evolutionary trajectories in blueberry and Arabidopsis [19,34,35].

### 3.3. VcUSPs Play Important Roles in Plant Responses to UV-B Radiation and ABA Treatments

USPs participate in a broad range of cellular responses to biotic and abiotic stress, and their roles in providing stress resistance in many plants have been reported. For instance, the overexpression of *MfUSP1* (*Medicago falcata*) resulted in increased tolerance to freezing, salinity, osmotic stress, and methyl viologen-induced oxidative stress [22]. The heterologous expression of *VvUSPA2*, *VvUSPA3*, *VvUSPA11*, *VvUSPA13*, and *VvUSPA16* in *E. coli* enhanced resistance to drought stress [19]. The overexpression of *AtUSP* (At3g53990) conferred strong tolerance to heat shock and oxidative stress in Arabidopsis [18]; however, AtUSP17 negatively regulates salt tolerance in Arabidopsis by modulating ethylene, ABA, reactive oxygen species, and G-protein signaling and responses [17]. Therefore, USP homologs may play different roles in plant stress responses.

UV-B radiation, an environmental signal, limits plant growth and development. In *E. coli*, the deletion of *UspA*, *UspC*, *UspD*, or *UspE* resulted in an enhanced sensitivity to UV-B exposure [47,48]. Microarray data show that most Arabidopsis *AtUSP* genes are induced by UV-B treatment [20]. In this study, we identified 21 *VcUSP* genes that were responsive to UV-B radiation based on transcriptome data. Most of these *VcUSP*s belong to groups Ⅰ and Ⅲ; group I *VcUSP*s were downregulated and group Ⅲ *VcUSP*s were upregulated in response to UV-B treatment. All group Ⅰ VcUSP proteins contained one UspA domain and one ATP-binding site and shared highly similar conserved motifs, while most group Ⅲ VcUSP proteins contained not only a UspA domain but also an UspF domain and similar conserved motifs. These results indicate that proteins within the same phylogenetic clade share close evolutionary relationships, conserved structures, and similar functions. Similar results have been obtained for other proteins, including MYBs and NACs [49,50].

The phytohormone ABA regulates plant responses to abiotic stress. In the wild tomato, *SpUSP* expression is markedly induced by ABA and plays important roles in drought tolerance by influencing ABA-induced stomatal movement [14]. Exogenously overexpressing *VyUSPA3* from Chinese wild grape improved drought tolerance in transgenic *V. vinifera*, likely by regulating the ABA signaling pathway [16]. Indeed, USP genes are induced by ABA in various plant species [19,22,36]. In the current study, we determined that only seven *VcUSP* genes responded to ABA treatment, including six upregulated and one (*VcUSP4* from group Ⅰ) downregulated gene. Most of the upregulated VcUSP genes (*VcUSP15*, *VcUSP16*, *VcUSP39*, and *VcUSP41*) showed a more than four-fold increase in expression compared to control conditions. Similar to other plant species, most differentially expressed *VcUSP*s in response to ABA were upregulated. Therefore, these genes might improve stress tolerance in blueberry by regulating the ABA signaling pathway. The large changes in expression of the seven DEGs in response to ABA treatment confirm the notion that these genes regulate ABA-related stress responses.

ABA mediates the core signaling network in the plant abiotic stress response [51,52]. Several studies showed that ABA treatment increased the tolerance of grapevine to UV-B radiation [23,26]; however, the evidence for a direct interaction between the UV-B and ABA pathways was only obtained for a few measured traits in Yunnan poplar (*Populus yunnanensis*) [24]. Which proteins are possibly involved in the interaction between ABA and UV-B signaling is unclear currently. Here, we found that both UV-B radiation and ABA treatments induced the expressions of *VcUSP41* and *VcUSP68*, with the former upregulated by both treatments and the latter upregulated by ABA but downregulated by UV-B. Therefore, it is possible that *VcUSP41* and *VcUSP68* act to bridge the UV-B and ABA signaling pathways for regulating stress responses or physiological and biochemical functions in blueberry.

### 3.4. Functional Analysis of VcUSPs under UV-B Radiation and ABA Treatments

USPs are small proteins that exist as monomers, dimers, trimers, and oligomers and regulate stress responses by interacting with various proteins [4,14,16,53]. In *Catharanthus roseus*, uspA-like transcripts are co-expressed with many putative ethylene-responsive bHLH or WRKY transcription factor genes [54]. In the current study, we used WGCNA to elucidate whether selected VcUSP proteins (VcUSP26, VcUSP67, VcUSP68, VcUSP22, and VcUSP41) possibly interact with transcription factors, such as MYB, WRKY, AP2, zinc finger, bHLH, auxin, and AUX/IAA family members, under UV-B or ABA treatment. KEGG pathway annotation showed that these transcription factors were mainly involved in the plant MAPK signaling pathway, plant–pathogen interactions, plant hormone signal transduction, and circadian rhythms (Tables S6–S8). WRKY transcription factors regulate physiological programs including pathogen defense, senescence, and the MAPK signaling pathway [55,56,57,58]. The auxin-responsive protein IAA (IAA or AUX) and auxin response factors (ARFs) are associated with the auxin signaling pathway [59], while ABA-INSENSITIVE 5 (ABI5) is involved in the ABA signaling pathway [60]. *LATE ELONGATED HYPOCOTYL* (*LHY*) of the MYB family and *PSEUDO RESPONSE REGULATOR* (*PRR*) form an early feedback loop in the circadian clock [61,62]. In Arabidopsis, USP regulates the circadian rhythm of the central clock genes [21]. Exogenously overexpressing *VyUSPA3* from Chinese wild grape improved drought tolerance in transgenic *V. vinifera*, possibly by interacting with a phytohormone signaling pathway, an ubiquitination system, ethylene-responsive element binding factors, or nuclear factors [16]. Thus, *VcUSP26*, *VcUSP67*, *VcUSP68*, *VcUSP22*, and *VcUSP41* may be involved in the UV-B or ABA-induced MAPK signaling pathways, plant–pathogen interactions, plant hormone signal transduction, and circadian rhythms.

### 3.5. VcUSPs May Be Involved in UV-B-Induced Flavonoid Biosynthesis

Plants are typically subjected to UV-B radiation, which activates UV RESISTANCE LOCUS 8 (UVR8) to interact with the E3 ubiquitin ligase CONSTITUTIVE PHOTOMORPHOGENIC 1 (COP1) [63,64]. ELONGATED HYPOCOTYL 5 (HY5) acts downstream of the UV-B photoreceptor UVR8 to regulate the expression of *MYB12* in response to UV-B radiation [65,66], which regulates flavonol accumulation [67,68]. In this regulatory network, the B-box protein BBX21 directly binds to cis-elements in the *HY5* promoter to activate its expression and interacts with BBX32 [69,70]. We found that *VcUSP26*, *VcUSP67*, and *VcUSP68* were co-expressed with *COP1*, *BBX21*, *BBX32*, and *MYB12*, while *VcUSP22* and *VcUSP41* were co-expressed with *HY5* under UV-B treatment. At the same time, *VcUSP26*, *VcUSP6*7, and *VcUSP68* were co-expressed with *MYB114* (*AtMYB114* homolog), *MYBA* (*AtTT2* homolog), *MYB11* (*AtMYB114* homolog), *MYB12* (*AtMYB12* homolog), and *MYBPA* (*AtMYB5* homolog) under UV-B radiation; these co-expressed genes control the biosynthesis of flavonoids, including anthocyanins, proanthocyanidins, and flavonols [71,72,73,74]. These finding suggest that VcUSP26, VcUSP67, VcUSP68, VcUSP22, and VcUSP41 function in the network involved in UV-B-induced flavonoid biosynthesis.

## 4. Materials and Methods

### 4.1. Identification of Putative VcUSPs 

To identify putative *VcUSP*s, Hidden Markov Model searches were performed in the Genome Database for the *Vaccinium corymbosum* cv. Draper V1.0 genome sequence (https://www.vaccinium.org/ (accessed on 1 July 2023)) using the USP domain (PF00582) from the Pfam database (http://pfam.xfam.org/ (accessed on 1 July 2023)) as a query. The candidate *VcUSP*s were investigated using the online programs Pfam and CDD (https://www.ncbi.nlm.nih.gov/Structure/cdd/wrpsb.cgi (accessed on 2 July 2023)), and genes without USP or AANH domains in their encoded proteins were removed. Finally, a list of *VcUSP*s with at least one USP domain was obtained by deleting redundant sequences based on sequence alignments generated using DNAMAN version 6.0.3.99 (Lynnon Biosoft, San Ramon, CA, USA). The molecular weight, theoretical isoelectric point (pI), instability index, aliphatic index, and grand average of hydropathicity of the proteins were calculated for the VcUSP proteins using the online program ExPASy (https://web.expasy.org/protparam/ (accessed on 4 July 2023)).

### 4.2. Phylogenetic Analysis and Multiple Sequence Alignment

The deduced amino acid sequences of the VcUSPs were aligned using DNAMAN version 6.0.3.99. The USP amino acid sequences of *A. thaliana* and *V. vinifera* were downloaded from NCBI (https://www.ncbi.nlm.nih.gov/ (accessed on 5 July 2023)). The phylogenetic trees were constructed in MEGA X version 11.0.10 (https://www.megasoftware.net/ (accessed on 2 July 2023)) using the Maximum Likelihood method. A bootstrap analysis was carried out with 1000 replicates [75].

### 4.3. Analysis of the Major Characteristics of VcUSP Family Members 

The amino acid sequences of the VcUSPs were subjected to a BLAST search against the NCBI database to predict the conserved UspA, UspE, and UspF domains. The conserved domains used for visualization were predicted using the online program CDD (https://www.ncbi.nlm.nih.gov/Structure/bwrpsb/bwrpsb.cgi (accessed on 10 July 2023)). Generic feature format files of the *VcUSP* family members were downloaded from the Genome Database for *Vaccinium*, including sequence information for the untranslated regions (UTRs), exons, and introns. The conserved motifs of the VcUSP amino acid sequences were uploaded to the online search tool MEME (http://meme-suite.org/tools/meme (accessed on 10 July 2023)), with the maximum number of motifs set at 20 and the order of site distribution set to zero or one occurrence per sequence. To analyze the cis-regulatory elements in the promoters of the *VcUSP*s, the 2000 bp upstream sequence of each gene was downloaded from the Genome Database for *Vaccinium* and submitted to the online program PlantCARE (https://bioinformatics.psb.ugent.be/webtools/plantcare/html/ (accessed on 20 July 2023)). The conserved domains, conserved motifs, exon–intron structures, and cis-regulatory elements in the promoters of the *VcUSP*s were visualized using TBtools-Ⅱ software (version 2.003) [76].

### 4.4. Differentially Expressed VcUSPs under UV-B Radiation or ABA Treatment 

Differentially expressed *VcUSP* genes under 311 nm UV-B radiation (0, 1, 3, 6, 12, or 24 h) or 100 µM ABA treatment (0, 6, or 12 h) were downloaded from the BioProject database in the NCBI repository (https://www.ncbi.nlm.nih.gov/bioproject; accession numbers PRJNA831018 and PRJNA997066, respectively. (accessed on 1 Auguest 2023)) [77]. The DEGs were identified by comparing the ABA-treated samples with the control (0 h) sample based on the FPKM (Fragments per Kilobase of transcript per Million mapped reads) values using the criteria of absolute log2(fold change) ≥ 1 and false discovery rate (FDR) < 0.01 performed using DESeq2 [78]. A heatmap of DEGs based on log10 (FPKM) values under UV-B radiation (0, 1, 3, 6, 12, or 24 h) or ABA treatment (0, 6, or 12 h) was constructed with TBtools-Ⅱ software (version 2.003).

### 4.5. WGCNA

WGCNA was performed on transcriptome data obtained from plants under UV-B radiation or ABA treatment using the WGCNA package in R [79]. The hierarchical clustering tree was built based on the correlation coefficients of different nodes. The different branches of the clustering tree represent different gene modules. Genes with different expression levels were assigned to various modules using the Dynamic Tree Cut R package. Since the degree of co-expression is high for genes in the same modules, *VcUSP*s and transcription factor genes from the same module were screened. Pearson’s correlation coefficient (*r*) analysis was performed between VcUSPs and transcription factor genes according to the FPKM values using SPSS 19.0 software (IBM, Armonk, NY, USA). Pairs of genes with a *p* value ≤ 0.05 were considered to be significantly correlated. The co-expression networks were visualized based on their *r* values using Cytoscape version 3.9.1 [80]. The KEGG [81], NCBI non-redundant protein sequences (NR) [82], and Protein family (Pfam) [83] databases, as well as a manually annotated and reviewed protein sequence database (Swiss-Prot) [84] and evolutionary genealogy of genes, Non-supervised Orthologous Groups (eggNOG) [85], were used to screen the transcription factor genes and predict their biological functions.

### 4.6. Validation of RNA-Seq Data Using RT-qPCR

The blueberry cultivar ‘Northland’ calli and in vitro–grown seedlings were used for UV-B radiation and ABA treatments, respectively. The blueberry calli and in vitro–grown seedlings were cultured on modified woody plant medium (WPM) containing Murashige and Skoog vitamins with 3.0 mg/L 2,4-dichlorophenoxyacetic acid (calli) or 1.0 mg/L trans-Zeatin (seedlings) under a 16 h light/8 h dark photoperiod at 25 °C and subcultured every 3 weeks (calli) and 5 weeks (seedlings). UV-B was applied by the means of narrow band lamps (TL20/01; 311 nm Philips, Amsterdam, Netherlands) positioned above the calli at the height of about 10 cm for 0, 1, 3, 6, 12, or 24 h [77]. The seedlings with ten to twelve blades were transferred to the medium containing 100 µM ABA for 0, 6 or 12 h. The calli or seedlings were harvested right after treatments of UV-B radiation or ABA and frozen in liquid nitrogen and stored at −80 °C for RT-qPCR analysis.

Total RNA was extracted from each sample subjected to UV-B radiation (0, 1, 3, 6, 12, or 24 h) or 100 µM ABA treatment (0, 6, or 12 h) using an RNA Extraction Kit (Sangon Biotech, Shanghai, China), and first-strand cDNAs were synthesized using PrimeScript RT Master Mix (Takara Bio, Kusatsu, Japan). Six differentially expressed *VcUSP* genes under UV-B radiation (*VcUSP1*, *VcUSP5*, *VcUSP13*, *VcUSP41*, *VcUSP51*, and *VcUSP68*) and seven under ABA treatment (*VcUSP4*, *VcUSP11*, *VcUSP15*, *VcUSP16*, *VcUSP39*, *VcUSP41*, and *VcUSP68*) were subjected to RT-qPCR analysis using an ABI 7900HT Real-time PCR system (Thermo Fisher Scientific, Waltham, MA, USA). Glyceraldehyde-3-phosphate dehydrogenase (*GAPDH*; GenBank accession no. AY123769) was used as the reference gene; the primer sequences are shown in Supplementary Table S10. The relative expression level of each gene was calculated using the 2^−∆∆Ct^ method for RT-qPCR analysis and the fold change method for the RNA-seq data. All experiments were carried out with three independent biological replicates, and three technical replicates were performed for each biological replicate. Tukey’s test was used to identify significant differences at *p* value ≤ 0.05 using SPSS 19.0 software.

## 5. Conclusions

In conclusion, a total of 72 putative *VcUSP* genes were identified and classified into five groups, in which 21 *VcUSP*s responded to UV-B radiation and 7 responded to exogenous ABA, and *VcUSP41* and *VcUSP68* might act as bridges integrating UV-B and ABA signaling. WGCNA predicted that VcUSP22, VcUSP41, VcUSP26, VcUSP67, and VcUSP68 may be involved in plant hormone signal transduction, circadian rhythms, the MAPK signaling pathway, and UV-B-induced flavonoid biosynthesis under UV-B or ABA treatment. Our findings provide a useful reference for subsequent research investigating the biological function of VcUSP family members in blueberry.

## Figures and Tables

**Table 1:** Detailed information about VcUSP proteins.

Gene Name	Protein Length (aa)	MW ^1^ (kDa)	pI ^2^	USP Domains	Other Domains	ATP-Binding Site	Group ^9^
*VcUSP1*	162	17.85	5.71	UspA	--	ATP	Ⅰ
*VcUSP2*	404	43.48	5.31	UspA	PMEI-like_2 ^3^	ATP	Ⅰ
*VcUSP3*	229	25.38	8.9	UspA	--	ATP	Ⅰ
*VcUSP4*	432	46.37	5.35	UspA	PMEI-like_2	ATP	Ⅰ
*VcUSP5*	163	18.15	5.5	UspA	--	ATP	Ⅰ
*VcUSP6*	344	36.57	4.68	UspA	PMEI-like	ATP	Ⅰ
*VcUSP7*	355	37.67	4.62	UspA	PMEI-like	ATP	Ⅰ
*VcUSP8*	150	16.22	6.41	UspA	--	ATP	Ⅰ
*VcUSP9*	164	17.66	8.76	UspA	--	ATP	Ⅰ
*VcUSP10*	164	18.43	6.75	UspA	--	ATP	Ⅰ
*VcUSP11*	177	19.74	5.43	UspA	--	ATP	Ⅰ
*VcUSP12*	173	19.26	5.88	UspA	--	ATP	Ⅰ
*VcUSP13*	225	24.64	4.85	UspA	--	ATP	Ⅰ
*VcUSP14*	253	27.87	5.34	UspA	--	ATP	Ⅰ
*VcUSP15*	232	25.81	4.93	UspA	--	ATP	Ⅰ
*VcUSP16*	262	29.01	5.1	UspA	--	ATP	Ⅰ
*VcUSP17*	235	25.69	5.14	UspA	--	ATP	Ⅰ
*VcUSP18*	328	35.73	5.1	UspA	PME ^4^	ATP	Ⅰ
*VcUSP19*	296	32.24	4.99	UspA	--	ATP	Ⅰ
*VcUSP20*	321	35.13	5.72	UspA	--	ATP	Ⅰ
*VcUSP21*	251	27.24	5.21	UspA	--	ATP	Ⅰ
*VcUSP22*	249	27.41	8.68	UspA	--	No	Ⅱ
*VcUSP23*	234	25.68	7.66	UspA/UspE	--	No	Ⅱ
*VcUSP24*	170	18.58	5.3	UspA/UspE	--	ATP	Ⅱ
*VcUSP25*	173	18.55	8.14	UspA	--	No	Ⅱ
*VcUSP26*	183	20.33	5.96	UspA	--	ATP	Ⅱ
*VcUSP27*	219	24.3	6.16	UspA	--	ATP	Ⅱ
*VcUSP28*	180	20.1	5.86	UspA/UspE	--	ATP	Ⅱ
*VcUSP29*	265	28.58	5.88	UspA	--	No	Ⅱ
*VcUSP30*	170	18.04	6.95	UspA/UspF	--	No	Ⅱ
*VcUSP31*	268	29.76	6.07	UspA	--	No	Ⅱ
*VcUSP32*	466	50.22	6.72	UspA + UspA + UspA	--	2ATP	Ⅱ
*VcUSP33*	419	44.73	6.38	UspA + UspA + UspA	--	2ATP	Ⅱ
*VcUSP34*	347	37.45	7.35	UspA + UspA	--	ATP	Ⅱ
*VcUSP35*	524	56.94	6.17	UspA	RING_Ubox ^5^	No	Ⅱ
*VcUSP36*	561	61.16	6.25	UspA	RING_H2 ^6^	No	Ⅱ
*VcUSP37*	587	63.69	6.15	UspA	RING_Ubox	ATP	Ⅱ
*VcUSP38*	584	63.66	6.24	UspA	RING_Ubox	ATP	Ⅱ
*VcUSP39*	161	17.08	6.49	UspA/UspF	--	ATP	Ⅱ
*VcUSP40*	212	23.03	6.39	UspA/UspF	--	ATP	Ⅱ
*VcUSP41*	178	19.64	7.61	UspA/UspF	--	No	Ⅲ
*VcUSP42*	172	18.67	6.72	UspA/UspF	--	ATP	Ⅲ
*VcUSP43*	168	18.35	6.73	UspA/UspF	--	ATP	Ⅲ
*VcUSP44*	307	33.87	5.87	UspA/UspF + UspA/UspF	--	ATP	Ⅲ
*VcUSP45*	160	17.8	6.2	UspA	--	No	Ⅲ
*VcUSP46*	160	17.69	6.59	UspA/UspF	--	ATP	Ⅲ
*VcUSP47*	96	10.65	4.87	UspA	--	No	Ⅲ
*VcUSP48*	170	18.71	6.31	UspA/UspF	--	ATP	Ⅲ
*VcUSP49*	147	16.76	5.38	UspA/UspF	--	No	Ⅲ
*VcUSP50*	144	16.24	5.82	UspA/UspF	--	No	Ⅲ
*VcUSP51*	145	16.09	5.08	UspA/UspF	--	No	Ⅲ
*VcUSP52*	423	47.02	5.4	UspA/UspF + UspA + UspA/UspF	--	ATP	Ⅲ
*VcUSP53*	132	14.81	5.12	UspA/UspE	--	No	Ⅲ
*VcUSP54*	195	21.58	4.95	UspA + UspA	--	No	Ⅲ
*VcUSP55*	323	35.68	7.47	(UspA + UspA)/UspE	--	ATP	Ⅲ
*VcUSP56*	132	14.57	5.11	UspA/UspF	--	No	Ⅲ
*VcUSP57*	234	25.63	9.53	UspA	STK_N ^7^	No	Ⅳ
*VcUSP58*	208	22.73	9.17	UspA	STK_N	No	Ⅳ
*VcUSP59*	99	10.92	10.09	UspA	--	No	Ⅳ
*VcUSP60*	375	42.34	6.26	UspA	tolA ^8^	No	Ⅳ
*VcUSP61*	410	46.17	5.58	UspA	--	No	Ⅳ
*VcUSP62*	261	28.58	5.55	UspA	--	No	Ⅳ
*VcUSP63*	224	24.49	4.98	UspA	--	No	Ⅳ
*VcUSP64*	274	29.93	5.82	UspA	--	No	Ⅳ
*VcUSP65*	146	16.85	7	UspA	--	No	Ⅴ
*VcUSP66*	200	22.62	10.51	UspA	--	No	Ⅴ
*VcUSP67*	240	26.76	7.01	UspA	--	No	Ⅴ
*VcUSP68*	267	28.56	9.05	UspA	--	No	Ⅴ
*VcUSP69*	218	24.12	10.18	UspA	--	No	Ⅴ
*VcUSP70*	232	26.4	10.22	UspA	--	No	Ⅴ
*VcUSP71*	151	16.04	5.68	UspA	--	No	Ⅴ
*VcUSP72*	201	21.61	7.81	UspA	--	No	Ⅴ

^1^ Molecular weight. ^2^ Theoretical isoelectric point. ^3^ Invertase/pectin methylesterase inhibitor. ^4^ Pectinesterase/pectinesterase inhibitor. ^5^ RING finger domain and U-box domain superfamily. ^6^ Subclass H2 RING finger domain. ^7^ N-terminal domain of eukaryotic serine threonine kinases. ^8^ Cell envelope integrity inner membrane protein. ^9^ Groups I–V come from Figure 1.

## Data Availability

All data in this study are included in the Supplementary Materials.

## Supplementary Materials

The following supporting information can be downloaded at: https://www.mdpi.com/article/10.3390/ijms242316819/s1.

## References

[1] Luo D., Wu Z., Bai Q., Zhang Y., Huang M., Huang Y., Li X. (2023). Universal stress proteins: From gene to function. Int. J. Mol. Sci..

[2] Nachin L., Nannmark U., Nyström T. (2005). Differential roles of the universal stress proteins of *Escherichia coli* in oxidative stress resistance, adhesion, and motility. J. Bacteriol..

[3] Tkaczuk K.L., Shumilin A., Chruszcz M., Evdokimova A.S., Minor W. (2013). Structural and functional insight into the universal stress protein family. Evol. Appl..

[4] Nachin L., Brive L., Persson K., Svensson P., Nyström T. (2008). Heterodimer formation within universal stress protein classes revealed by an *in silico* and experimental approach. J. Mol. Biol..

[5] Nystrom T., Neidhardt F.C. (1992). Cloning, mapping and nucleotide sequencing of a gene encoding a universal stress protein in *Escherichia coli*. Mol. Microbiol..

[6] Freestone P., Nyström T., Trinei M., Norris V. (1997). The universal stress protein, UspA, of *Escherichia coli* is phosphorylated in response to stasis. J. Mol. Biol..

[7] Kvint K., Nachin L., Diez A., Nyström T. (2003). The bacterial universal stress protein: Function and regulation. Curr. Opin. Microbiol..

[8] Sousa M.C., McKay D.B. (2001). Structure of the universal stress protein of *Haemophilus influenzae*. Structure.

[9] Zarembinski T.I., Hung L.W., Mueller-Dieckmann H.J., Kim K.K., Yokota H., Kim R., Kim S.S. (1998). Structure-based assignment of the biochemical function of a hypothetical protein: A test case of structural genomics. Proc. Natl. Acad. Sci. USA.

[10] Aravind L., Anantharaman V., Koonin E.V. (2002). Monophyly of class I aminoacyl tRNA synthetase, USPA, ETFP, photolyase, and PP-ATPase nucleotide-binding domains: Implications for protein evolution in the RNA world. Proteins.

[11] Hassan S., Ahmad A., Batool F., Rashid B., Husnain T. (2021). Genetic modification of *Gossypium arboreum* universal stress protein (GUSP1) improves drought tolerance in transgenic cotton (*Gossypium hirsutum*). Physiol. Mol. Biol. Plants.

[12] Sauter M., Rzewuski G., Marwedel T., Lorbiecke R. (2002). The novel ethylene-regulated gene *OsUsp1* from rice encodes a member of a plant protein family related to prokaryotic universal stress proteins. J. Exp. Bot..

[13] Park S., Jung Y.J., Lee Y., Kim I.R., Seol M., Kim E., Jang M., Lee J.R. (2017). Functional characterization of the Arabidopsis universal stress protein AtUSP with an antifungal activity. Biochem. Biophys. Res. Commun..

[14] Loukehaich R., Wang T., Ouyang B., Ziaf K., Li H., Zhang J., Lu Y., Ye Z. (2012). *SpUSP*, an annexin-interacting universal stress protein, enhances drought tolerance in tomato. J. Exp. Bot..

[15] Yang M., Che S., Zhang Y., Wang H., Wei T., Yan G., Song W., Yu W. (2019). Universal stress protein in *Malus sieversii* confers enhanced drought tolerance. J. Plant Res..

[16] Cui X., Zhang P., Chen C., Zhang J. (2023). VyUSPA3, a universal stress protein from the Chinese wild grape *Vitis yeshanensis*, confers drought tolerance to transgenic *V. vinifera*. Plant Cell Rep..

[17] Bhuria M., Goel P., Kumar S., Singh A.K. (2022). AtUSP17 negatively regulates salt stress tolerance through modulation of multiple signaling pathways in *Arabidopsis*. Physiol. Plantarum.

[18] Jung Y.J., Melencion S.M.B., Lee E.S., Park J.H., Alinapon C.V., Oh H.T., Yun D., Chi Y.H., Lee S.Y. (2015). Universal stress protein exhibits a redox-dependent chaperone function in *Arabidopsis* and enhances plant tolerance to heat shock and oxidative stress. Front. Plant Sci..

[19] Cui X., Zhang P., Hu Y., Chen C., Liu Q., Guan P., Zhang J. (2021). Genome-wide analysis of the *Universal stress protein A* gene family in *Vitis* and expression in response to abiotic stress. Plant Physiol. Biochem..

[20] Bhuria M., Goel P., Kumar S., Singh A.K. (2019). Genome-wide identification and expression profiling of genes encoding universal stress proteins (USP) identify multi-stress responsive USP genes in *Arabidopsis thaliana*. Plant Physiol. Rep..

[21] Phan K.A.T., Paeng S.K., Chae H.B., Park J.H., Lee E.S., Wi S.D., Bae S.B., Kim M.G., Yun D., Kim W. (2022). Universal stress protein regulates the circadian rhythm of central oscillator genes in *Arabidopsis*. FEBS Lett..

[22] Gou L., Zhou C., Lu S., Guo Z. (2020). A Universal Stress Protein from *Medicago falcata* (*MfUSP1*) confers multiple stress tolerance by regulating antioxidant defense and proline accumulation. Environ. Exp. Bot..

[23] Berli F.J., Moreno D., Piccoli P., Hespanhol-Viana L., Silva M.F., Bressan-Smith R., Cavagnaro J.B., Bottini R. (2010). Abscisic acid is involved in the response of grape (*Vitis vinifera* L.) cv. Malbec leaf tissues to ultraviolet-B radiation by enhancing ultraviolet- absorbing compounds, antioxidant enzymes and membrane sterols. Plant Cell Environ..

[24] Duan B., Xuan Z., Zhang X., Korpelainen H., Li C. (2008). Interactions between drought, ABA application and supplemental UV-B in *Populus yunnanensis*. Physiol. Plantarum.

[25] Rakitin V.Y., Karyagin V.V., Rakitina T.Y., Prudnikova O.N., Vlasov P.V. (2008). UV-B stress-induced ABA production in *Arabidopsis thaliana* mutants defective in ethylene signal transduction pathway. Russ. J. Plant Physiol..

[26] Berli F.J., Fanzone M., Piccoli P., Bottini R. (2011). Solar UV-B and ABA are involved in phenol metabolism of *Vitis vinifera* L. increasing biosynthesis of berry skin polyphenols. J. Agric. Food Chem..

[27] Tran P.H.L., Tran T.T.D. (2021). Blueberry supplementation in neuronal health and protective technologies for efficient delivery of blueberry anthocyanins. Biomolecules.

[28] Wang W., Guo Y., Liu M., Chen X., Xiao X., Wang S., Gong P., Ma Y., Che F. (2022). Structure and function of blueberry anthocyanins: A review of recent advances. J. Funct. Foods.

[29] Nguyen C.T.T., Lim S., Lee J.G., Lee E.J. (2017). VcBBX, VcMYB21, and VcR2R3MYB transcription factors are involved in UV-B-induced anthocyanin biosynthesis in the peel of harvested blueberry fruit. J. Agric. Food Chem..

[30] Oh H.D., Yu D.J., Chung S.W., Chea S., Lee H.J. (2018). Abscisic acid stimulates anthocyanin accumulation in ‘Jersey’ highbush blueberry fruits during ripening. Food Chem..

[31] González-Villagra J., Marjorie R., Alberdi M., Acevedo P., Loyola R., Tighe-Neira R., Arce-Johnson P. (2020). Solar UV irradiation effects on photosynthetic performance, biochemical markers, and gene expression in highbush blueberry (*Vaccinium corymbosum* L.) cultivars. Sci. Hortic..

[32] Karppinen K., Tegelberg P., Häggman H., Jaakola L. (2018). Abscisic acid regulates anthocyanin biosynthesis and gene expression associated with cell wall modification in ripening bilberry (*Vaccinium myrtillus* L.) fruits. Front. Plant Sci..

[33] Riechmann J.L., Heard J., Martin G., Reuber L., Jiang C., Keddie J., Adam L., Pineda O., Ratcliffe O.J., Samaha R.R. (2000). Arabidopsis transcription factors: Genome-wide comparative analysis among eukaryotes. Science.

[34] Kerk D., Bulgrien J., Smith D.W., Gribskov M. (2003). Arabidopsis proteins containing similarity to the universal stress protein domain of bacteria. Plant Physiol..

[35] Li W., Wei Y., Wang J., Liu C., Lan X., Jiang Q., Pu Z., Zheng Y. (2010). Identification, localization, and characterization of putative *USP* genes in barley. Theor. Appl. Genet..

[36] Arabia S., Sami A.A., Akhter S., Sarker R.H., Islam T. (2021). Comprehensive *in silico* characterization of universal stress proteins in rice (*Oryza sativa* L.) with insight into their stress-specific transcriptional modulation. Front. Plant Sci..

[37] Colle M., Leisner C.P., Wai C.M., Ou S., Bird K.A., Wang J., Wisecaver J.H., Yocca A.E., Alger E.I., Tang H. (2019). Haplotype-phased genome and evolution of phytonutrient pathways of tetraploid blueberry. GigaScience.

[38] Song Y., Ma B., Guo Q., Zhou L., Zhou X., Ming Z., You H., Zhang C. (2023). MYB pathways that regulate UV-B-induced anthocyanin biosynthesis in blueberry (*Vaccinium corymbosum*). Front. Plant Sci..

[39] Cannon S.B., Mitra A., Baumgarten A., Young N.D., May G. (2004). The roles of segmental and tandem gene duplication in the evolution of large gene families in *Arabidopsis thaliana*. BMC Plant Biol..

[40] Bahieldin A., Atef A., Shokry A.M., Al-Karim S., Attas S.G.A., Gadallah N.O., Edris S., Al-Kordy M.A., Omer A.M.S., Sabir J.S.M. (2015). Structural identification of putative USPs in *Catharanthus roseus*. Comptes Rendus Biol..

[41] Giovane A., Servillo L., Balestrieri C., Raiola A., D’Avino R., Tamburrini M., Ciardiello M.A., Camardella L. (2004). Pectin methylesterase inhibitor. Biochim. Biophys. Acta.

[42] Das A., Liang Y., Mariano J., Li J., Huang T., King A., Tarasov S.G., Weissman A.M., Ji X., Byrd R.A. (2013). Allosteric regulation of E2:E3 interactions promote a processive ubiquitination machine. EMBO J..

[43] Joo H., Lim C.W., Lee S.C. (2019). Roles of pepper bZIP transcription factor CaATBZ1 and its interacting partner RING-type E3 ligase CaASRF1 in modulation of ABA signalling and drought tolerance. Plant J..

[44] Lim S.D., Cho H.Y., Park Y.C., Ham D.J., Lee J.K., Jang C.S. (2013). The rice RING finger E3 ligase, OsHCI1, drives nuclear export of multiple substrate proteins and its heterogeneous overexpression enhances acquired thermo tolerance. J. Exp. Bot..

[45] Cozzone A.J. (1993). ATP-dependent protein kinase in bacteria. J. Cell Biochem..

[46] Levengood-freyermuth S.K., Click E.M., Webster R.E. (1993). Role of the carboxyl-terminal domain of TolA in protein import and integrity of the outer membrane. J. Bacteriol..

[47] Gustavsson N., Diez A.A., Nyström T. (2002). The universal stress protein paralogues of *Escherichia coli* are co-ordinately regulated and co-operate in the defence against DNA damage. Mol. Microbiol..

[48] Diez A., Gustavsson N., Nyström T. (2000). The universal stress protein A of *Escherichia coli* is required for resistance to DNA damaging agents and is regulated by a RecA/FtsK-dependent regulatory pathway. Mol. Microbiol..

[49] Dubos C., Stracke R., Grotewold E., Weisshaar B., Martin C., Lepiniec L. (2010). MYB transcription factors in *Arabidopsis*. Trends Plant Sci..

[50] Ooka H., Satoh K., Doi K., Nagata T., Otomo Y., Murakami K., Matsubara K., Osato N., Kawai J., Carninci P. (2003). Comprehensive analysis of NAC family genes in *Oryza sativa* and *Arabidopsis thaliana*. DNA Res..

[51] Cutler S.R., Rodriguez P.L., Finkelstein R.R., Abrams S.R. (2010). Abscisic acid: Emergence of a core signaling network. Annu. Rev. Plant Biol..

[52] Rozema J., van de Stanij J., Björn L.O., Caldwell M. (1997). UV-B as an environmental factor in plant life: Stress and regulation. Trends Ecol. Evol..

[53] Gutiérrez-Beltrán E., Personat J.M., de la Torre F., del Pozo O. (2017). A universal stress protein involved in oxidative stress is a phosphrylation target for protein kinase CIPK6. Plant Physiol..

[54] Bahieldin A., Atef A., Shokry A.M., Al-Karim S., Attas S.G.A., Gadallah N.O., Edris S., Al-kordy M.A., Hassan S.M., Abo-Aba S. (2017). Transcription factors regulating *uspA* genes in *Catharanthus roseus*. Comptes Rendus Biol..

[55] Rushton P.J., Somssich I.E., Ringler P., Shen Q.J. (2010). WRKY transcription factors. Trends Plant Sci..

[56] Xu X., Chen C., Fan B., Chen Z. (2006). Physical and functional interactions between pathogen-induced Arabidopsis WRKY18, WRKY40, and WRKY60 transcription factors. Plant Cell.

[57] Eulgem T., Somssich I.E. (2007). Networks of WRKY transcription factors in defense signaling. Curr. Opin. Plant Biol..

[58] Eulgem T., Rushton P.J., Robatzek S., Somssich I.E. (2000). The WRKY superfamily of plant transcription factors. Trends Plant Sci..

[59] Wang Y., Wang N., Xu H., Jiang S., Fang H., Su M., Zhang Z., Zhang T., Chen X. (2018). Auxin regulates anthocyanin biosynthesis through the Aux/IAA-ARF signaling pathway in apple. Hortic. Res..

[60] Chen K., Li G., Bressan R.A., Song C., Zhu J., Zhao Y. (2020). Abscisic acid dynamics, signaling, and functions in plants. J. Integr. Plant Biol..

[61] Nakamichi N., Kiba T., Henriques R., Mizuno T., Chua N., Sakakibara H. (2010). PSEUDO-RESPONSE REGULATORS 9, 7, and 5 are transcriptional repressors in the Arabidopsis circadian clock. Plant Cell.

[62] Wang L., Kim J., Somers D.E. (2013). Transcriptional corepressor TOPLESS complexes with pseudoresponse regulator proteins and histone deacetylases to regulate circadian transcription. Proc. Natl. Acad. Sci. USA.

[63] Rizzini L., Favory J.J., Cloix C., Faggionato D., O’Hara A., Kaiserli E., Baumeister R., Schäfer E., Nagy F., Jenkins G.I. (2011). Perception of UV-B by the Arabidopsis UVR8 protein. Science.

[64] Favory J., Stec A., Gruber H., Rizzini L., Oravecz A., Funk M., Albert A., Cloix C., Jenkins G.I., Oakeley E.J. (2009). Interaction of COP1 and UVR8 regulates UV-B-induced photomorphogenesis and stress acclimation in *Arabidopsis*. EMBO J..

[65] Bhatia C., Gaddam S.R., Pandey A., Trivedi P.K. (2021). COP1 mediates light-dependent regulation of flavonol biosynthesis through HY5 in Arabidopsis. Plant Sci..

[66] Stracke R., Favory J., Gruber H., Bartelniewoehner L., Bartels S., Binkert M., Funk M., Weisshaar B., Ulm R. (2010). The *Arabidopsis* bZIP transcription factor HY5 regulates expression of the PFG1/MYB12 gene in response to light and ultraviolet-B radiation. Plant Cell Environ..

[67] Mehrtens F., Kranz H., Bednarek P., Weisshaar B. (2005). The Arabidopsis transcription factor MYB12 is a flavonol-specific regulator of phenylpropanoid biosynthesis. Plant Physiol..

[68] Luo J., Butelli E., Hill L., Parr A., Niggeweg R., Bailey P., Weissharr B., Martin C. (2008). AtMYB12 regulates caffeoyl quinic acid and flavonol synthesis in tomato: Expression in fruit results in very high levels of both types of polyphenol. Plant J..

[69] Holtan H.E., Bandong S., Marion C.M., Adam L., Tiwari S., Shen Y., Maloof J.N., Maszle D.R., Ohto M., Preuss S. (2011). BBX32, an Arabidopsis B-Box protein, functions in light signaling by suppressing HY5-regulated gene expression and interacting with STH2/BBX21. Plant Physiol..

[70] Xu D., Jiang Y., Li J., Lin F., Holm M., Deng X.W. (2016). BBX21, an *Arabidopsis* B-box protein, directly activates *HY5* and is targeted by COP1 for 26S proteasome-mediated degradation. Proc. Natl. Acad. Sci. USA.

[71] Gonzalez A., Zhao M., Leavitt J.M., Lloyd A.M. (2007). Regulation of the anthocyanin biosynthetic pathway by the TTG1/bHLH/Myb transcriptional complex in Arabidopsis seedlings. Plant J..

[72] Baudry A., Heim M.A., Dubreucq B., Caboche M., Weisshaar B., Lepiniec L. (2004). TT2, TT8, and TTG1 synergistically specify the expression of *BANYULS* and proanthocyanidin biosynthesis in *Arabidopsis thaliana*. Plant J..

[73] Stracke R., Jahns O., Keck M., Tohge T., Niehaus K., Fernie A.R., Weisshaar B. (2010). Analysis of PRODUCTION OF FLAVONOL GLYCOSIDES-dependent flavonol glycoside accumulation in Arabidopsis thaliana plants reveals MYB11-, MYB12- and MYB111-independent flavonol glycoside accumulation. New Phytol..

[74] Gonzalez A., Mendenhall J., Huo Y., Lloyd A. (2009). TTG1 complex MYBs, MYB5 and TT2, control outer seed coat differentiation. Dev. Biol..

[75] Tamura K., Stecher G., Kumar S. (2021). MEGA 11: Molecular evolutionary genetics analysis version 11. Mol. Biol. Evol..

[76] Chen C., Chen H., Zhang Y., Thomas H.R., Frank M.H., He Y., Xia R. (2020). TBtools: An integrative toolkit developed for interactive analyses of big biological data. Mol. Plant.

[77] Song Y., Ma B., Guo Q., Zhou L., Lv C., Liu X., Wang J., Zhou X., Zhang C. (2022). UV-B induces the expression of favonoid biosynthetic pathways in blueberry (*Vaccinium corymbosum*) calli. Front. Plant Sci..

[78] Love M.I., Huber W., Anders S. (2014). Moderated estimation of fold change and dispersion for RNA-seq data with DESeq2. Genome Biol..

[79] Langfelder P., Horvath S. (2008). WGCNA: An *R* package for weighted correlation network analysis. BMC Bioinf..

[80] Shannon P., Markiel A., Ozier O., Baliga N.S., Wang J.T., Ramage D., Amin N., Schwikowski B., Ideker T. (2003). Cytoscape: A software environment for integrated models of biomolecular interaction networks. Genome Res..

[81] Kanehisa M., Goto S., Kawashima S., Okuno Y., Hattori M. (2004). The KEGG resource for deciphering the genome. Nucleic Acids Res..

[82] Deng Y., Li J., Wu S., Zhu Y., Chen Y., Fuchu H.E. (2006). Integrated nr database in protein annotation system and its localization. Comput. Eng..

[83] Finn R.D., Bateman A., Clements J., Coggill P., Eberhardt R.Y., Eddy S.R., Andreas H., Kirstie H., Lilisa H., Jaina M. (2014). Pfam: The protein families database. Nucleic Acids Res..

[84] Apweiler R., Bairoch A., Wu C.H., Barker W.C., Boeckmann B., Ferro S., Gasteiger E., Huang H., Lopez R., Magrane M. (2004). UniProt: The universal protein knowledgebase. Nucleic Acids Res..

[85] Huerta-Cepas J., Forslund K., Coelho L.P., Szklarczyk D., Jensen L.J., von Mering C., Bork P. (2017). Fast genome-wide functional annotation through orthology assignment by eggNOG-mapper. Mol. Biol. Evol..

