# Supplementary material for: Genome-Wide Analysis of the Universal Stress Protein Gene Family in Blueberry and Their Transcriptional Responses to UV-B Irradiation and Abscisic Acid
Source: Int J Mol Sci. 2023 Nov 27;24(23):16819. doi: 10.3390/ijms242316819 (PMC10706445; doi:10.3390/ijms242316819)
Supplement: Supplementary file 1 [file ijms-24-16819-s001.zip › Supplement Figures.pdf]

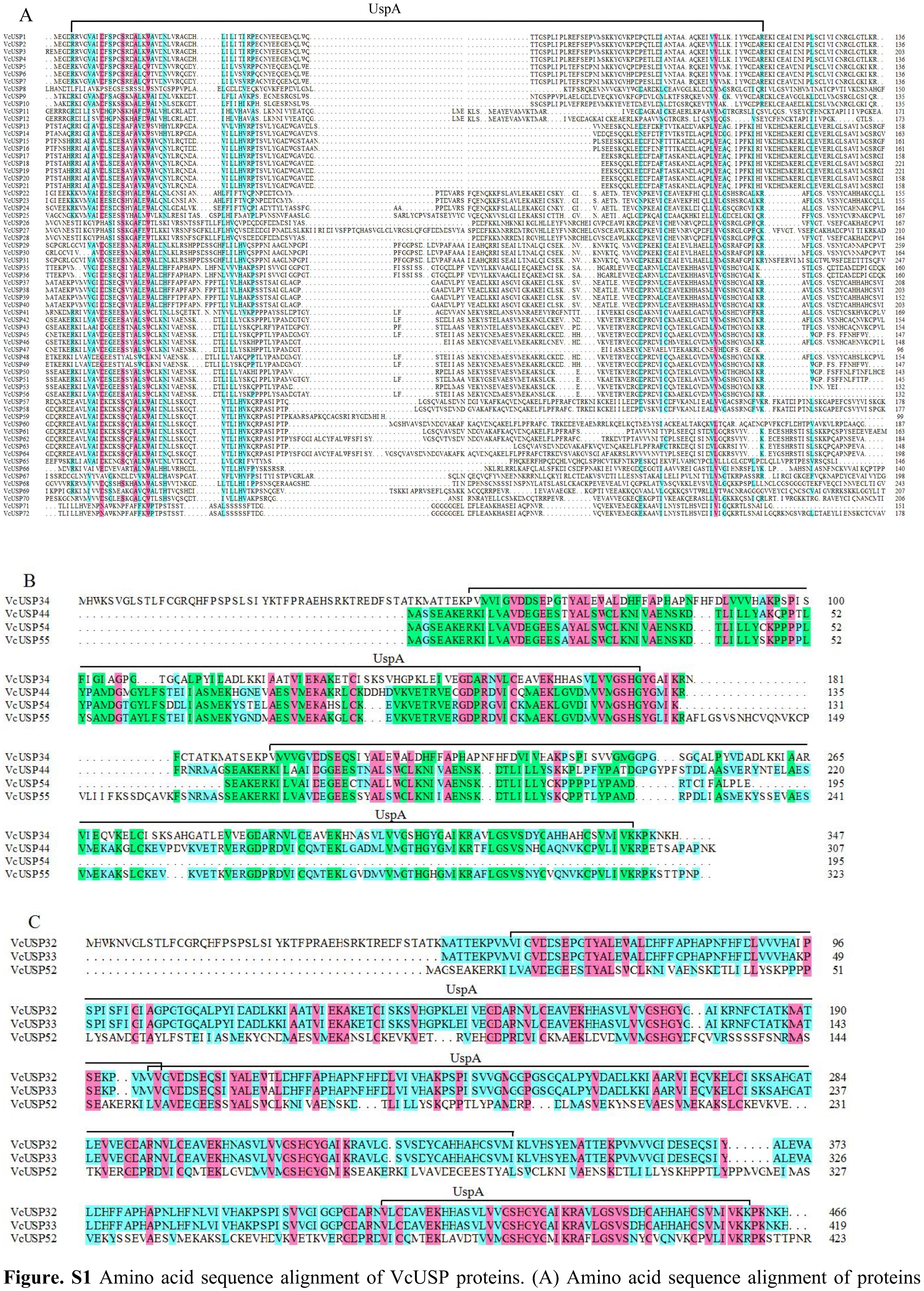

Figure. S1 Amino acid sequence alignment of VcUSP proteins. (A) Amino acid sequence alignment of proteins with one UspA domain. (B) Amino acid sequence alignment of proteins with two UspA domains. (C) Amino acid sequence alignment of proteins with three UspA domains. The pink background indicates 100% conservation; green indicates ⩾ 75% conservation; blue indicates ⩾ 50% conservation.

A B

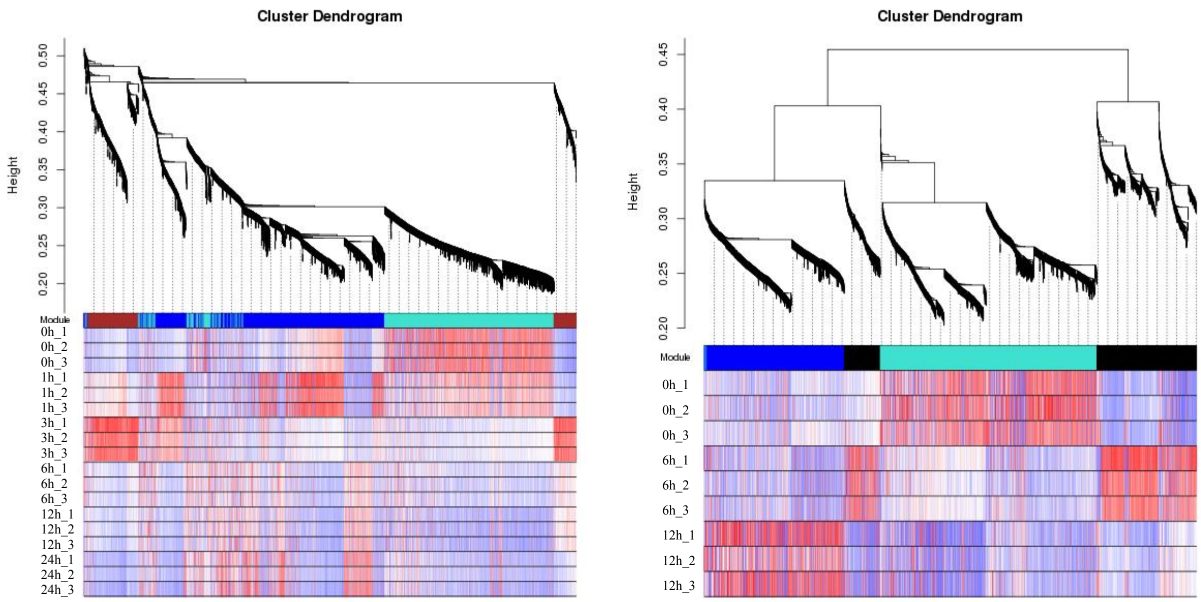

Figure. S2 Hierarchical clustering tree of the co-expression modules identified by WGCNA under UV-B radiation (**A**) and exogenous ABA (**B**) treatment. Different modules are marked by different colors. Each leaf of the cluster tree represents a gene. The darker the color in the corresponding heat map, the higher the correlation between the gene in the module and the treatment.

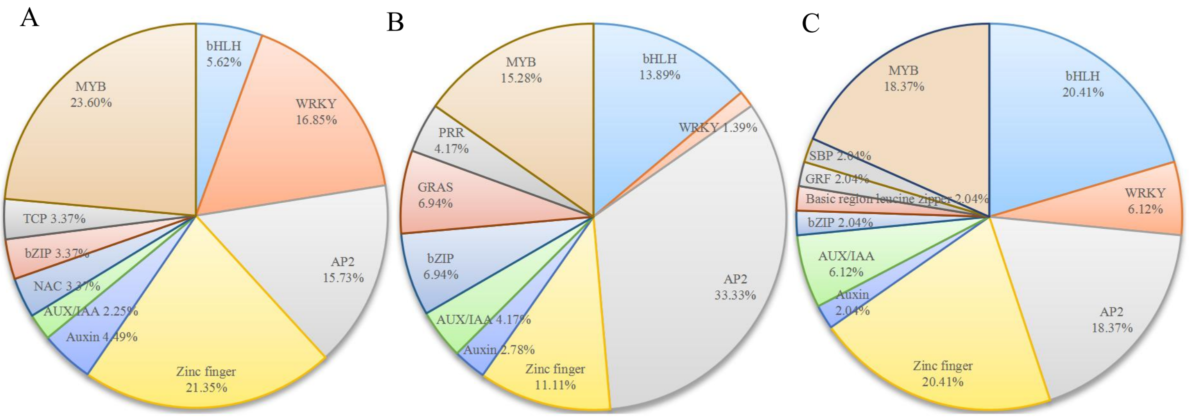

Figure. S3 Transcription factor family clusters under UV-B radiation or exogenous ABA treatment according to WGCNA. **A** The blue module shows transcription factor family clusters under UV-B radiation. **B** The turquoise module shows transcription factor family clusters under UV-B radiation. **C** The blue module shows transcription factor family clusters under exogenous ABA treatment.
